# Violence against women and children in the Pacific: A systematic scoping review and expert consultation of prevention interventions

**DOI:** 10.1371/journal.pgph.0006587

**Published:** 2026-07-02

**Authors:** Hattie Lowe, Fiona Langridge, Arieta Matalomani, Jurgita Seraikaite, Lucy Stevens, Louisa Apelu, Helen Tanielu, Jenevieve Mannell

**Affiliations:** 1 Institute for Global Health, University College London (UCL), London, United Kingdom; 2 Department of Paediatrics: Child and Youth Health, University of Auckland, Auckland, New Zealand; 3 Inclusive and Equitable Societies, Secretariat of the Pacific Community (SPC), Suva, Fiji; 4 Ending Violence Against Women and Girls, UN Women Pacific, Suva, Fiji; 5 UNDP Samoa, Apia, Samoa; 6 Faculty of Arts, National University of Samoa (National University of Samoa and Spotlight Initiative), Apia, Samoa; Yale University, UNITED STATES OF AMERICA

## Abstract

The Pacific region experiences some of the world’s highest rates of violence against women and children (VAW/C), yet it has also shown leadership and innovation in prevention efforts. Despite progress, evidence gaps remain, particularly regarding intervention impact. This scoping review systematically maps current evidence on VAW/C prevention in the Pacific, complemented by expert consultation. Eleven interventions were identified across five countries – Solomon Islands, Samoa, Aotearoa New Zealand, Fiji, and Papua New Guinea. Most interventions focused on VAW or family violence, with only one directly addressing VAC. Interventions were grouped into four categories based on their mechanisms of action: participatory learning and action, community mobilisation, community response, and policy or institutional response. Several interventions demonstrated promising outcomes, including reductions in VAW, shifts in norms, and increased community engagement. Interventions grounded in Pacific values and knowledge systems showed particularly strong potential. However, most were small in scale, pilot in nature, and assessed over short periods, limiting insight into mechanisms of change, sustainability and scalability. Despite gaps, the diversity and innovation of prevention efforts in the Pacific reflects growing momentum. Realising their potential will require sustained investment, local capacity strengthening, and coordinated implementation of culturally relevant, evidence-informed interventions and evaluation frameworks.

## Introduction

The Pacific region faces some of the highest global prevalence rates of violence against women (VAW) and children (VAC). Recent estimates suggest that 49% of women have experienced physical or sexual intimate partner violence (IPV) in their lifetime, the most common type of VAW [1]. Regional estimates for VAC are limited, but the available evidence points towards high rates of child maltreatment, violent discipline and childhood sexual abuse across Pacific countries [2,3]. VAC and VAW are inextricably linked, with strong evidence for exposure to violence in childhood predicting violence experience and perpetration in later life, globally, and in the Pacific [4–6]. VAC and VAW share similar risk factors and are reproduced through intergenerational cycles [7]. Consequently, there is a need for a coordinated and integrated approach to preventing both VAW and VAC. This is particularly true for the Pacific context where community- and family-centred approaches to prevention are critical given the communal and kin-based ways of living that are widely embedded and practised throughout the region [8,9].

The Pacific region is often mischaracterised in global narratives as remote and culturally homogeneous [8,10]. Such reductionist views of the Pacific are harmful for violence prevention efforts – historically, the Pacific has been underfunded and under-prioritised in the global VAW/C prevention agenda. In reality, the region spans nearly one-third of the Earth’s surface and is marked by significant linguistic, cultural, political and economic diversity [8,10,11]. Yet, the Pacific is often obscured from global accounts of VAW prevention efforts. For example, the £25 million global What Works to Prevent VAW initiative which began in 2014 focused on Africa, Asia and the Middle East [12], and the second What Works initiative launched in 2022 with a focus on scale up similarly does not include any work in the Pacific region. Equally, much of the VAC prevention work in the Global South has taken place in sub-Saharan Africa and South Asia [13–15].

Despite this lack of global attention, there has been growing action in the Pacific on tackling gender inequality and VAW/C over the past 50 years, laying the groundwork for future progress in the region. The Fiji Women’s Crisis centre established in the 1980s and the Pacific Network Against Violence Against Women and Girls were early pioneers in this work. A foundational step towards understanding the extent and drivers of VAW in the region has been through the collection of population-level data. For example, the Family Health and Safety Studies and DHS Multiple Indicator Cluster Surveys conducted across numerous Pacific countries have provided critical insight into the prevalence and drivers of VAW in the region, generating robust evidence to guide national policy and advocacy efforts [16,17]. These surveys have estimated high lifetime prevalence rates of intimate partner violence in the region, such as 53% in Kiribati, 52% in Fiji and 50% in the Solomon Islands, drawing global attention and urgency to the issue [1].

Multilateral organisations, particularly under the United Nations (UN) umbrella, have provided extensive economic investment into VAWC prevention, with programmes led by UN Women, UNDP, UNICEF, UNESCO and UNFPA working towards enhancing service quality and strengthening legal systems, advocacy and awareness raising. Specifically, the UN Sustainable Development Goal (SDG) programme has been pivotal in accelerating efforts to end VAW/C in the Pacific. Donors and development partners, such as the Australian Government Department for Foreign Affairs and Trade (DFAT) and the European Union (EU) have also invested heavily in this space. DFAT are a major funder of UN Women’s work in the region. The Global Spotlight Initiative, a three-year programme supported by the UN and EU, invested resources into institutional strengthening and capacity building, focusing on enhancing legal frameworks, raising public awareness, improving data collection systems and strengthening civil society organisations for the prevention of violence against women and girls (VAWG) in the Pacific [18]. The Spotlight Initiative worked in Fiji, Papua New Guinea, Samoa and Vanuatu and the Pacific Regional Programme also included Timor Leste, the Solomon Islands and Marshall Islands [19]. This represents the first time that multiple Pacific nations have been included within global VAW initiatives. Also funded by the EU, the Pacific Partnership to End VAWG (PPEVAWG), a multi-stakeholder regional initiative, aims to address the systemic drivers of gender-based violence through integrated approaches in education, service provision, institutional capacity-building, and normative change across Pacific Island countries [20]. Complementing these efforts, the UNICEF Pacific Child Protection Programme (2018–2023) worked across 14 Pacific Island Countries and Territories to strengthen national child protection systems, promote family and community-based prevention approaches, and support legislative reform and service delivery for the protection of children from violence, abuse and neglect [21].

Similarly, national action plans have emerged across the Pacific, such as the Fiji National Action Plan to Prevent Violence Against All Women and Girls from 2023 [22], the Solomon Islands National Action Plan on Women, Peace and Security in 2017 [23], and Samoa’s Pola Puipui National Prevention Framework which was launched in 2024 [24]. These initiatives represent a collective effort to embed VAW/C prevention into national governance structures and ensure it is prioritised and funded. Also at the national level the Pacific has seen the implementation of Family Protection Acts across numerous countries and reviews of existing legislation, such as the Samoa Law Reform Commission’s review of family laws in 2022 [25], marking important milestones in legislative efforts to protect women and children from violence in the region.

Finally, important regional networks and partnerships have been developed, such as the Secretariat of the Pacific Community’s (SPC) Regional Working Group (RWG) on the Implementation of Family Violence/ Family Protection Acts; the PPEVAWG Social Citizenship Education programme; the CSO-led Pacific Women’s Network Against VAW, and the Toksave gender resource platform amongst many others, which have supported data-sharing, knowledge exchange and cross-country action and advocacy [26–28].

Despite the considerable investments and extensive programming undertaken across the Pacific to address VAW/C, substantial gaps remain in our understanding of prevention and if and how existing programmes are working for the region. This is not because the programming is absent, but because there are few in-depth and large-scale evaluations of existing work that fit the criteria of rigour commonly prioritised in global health evidence hierarchies, which emphasise experimental or quasi-experimental designs such as randomised-controlled trials as the gold standard for demonstrating intervention effectiveness [29]. This may in part be due to regional resource and capacity constraints, as well as competing priorities. Ultimately, this limits our ability to assess the types of interventions and good practices that should be prioritised for scale up from a global health perspective. The Pacific demonstrates strong leadership and innovation in the prevention of VAW/C, yet much of the existing work has not been captured in ways that are recognised within these global health evidence hierarchies. This disconnect not only risks reinforcing existing inequities in global health knowledge production, but also limits opportunities for learning and adaptation both within and beyond the Pacific.

This review aims to address this gap by taking a more open and flexible approach to mapping the evidence on the interventions that have been implemented for VAW/C prevention. Our review aims to serve as a tool for Pacific researchers, practitioners, and policymakers to advocate and apply for funding and resources to conduct further evaluation in the region, capitalising on the current political will to tackle VAW/C demonstrated through recent work by governments, multilateral and civil society organisations and development partners. It also has the potential to contribute to growing calls to action for global health research to broaden its understanding of what constitutes rigorous evidence, by recognising and valuing alternative epistemologies and methodologies for intervention and evaluation that are more closely aligned with Pacific ways of knowing and doing [8]. This review is guided by two questions: 1) What evidence is available on the interventions that have been implemented to reduce VAW/C among Pacific populations?; and 2) What gaps in evidence remain for the prevention of VAW/C in Pacific populations?

## Methodology

We selected a scoping review method [30] to map relevant interventions that have been evaluated for the prevention of VAW/C in the Pacific region. This method was selected for its ability to capture a breadth of evidence, evaluation approaches (qualitative, quantitative, mixed methods), and the inclusion of grey literature from non-governmental organisations, religious entities, and regional networks – where a vast resource of relevant literature in the Pacific is produced and circulates. For the purposes of this study, the Pacific region is defined as the 22 Pacific Nations and Territories that constitute the Pacific Community [31]. We also include interventions conducted with Pacific populations in New Zealand and Australia, reflecting the significant diaspora of Pacific populations in these countries. We attempted to align our methods with Pacific epistemologies based in relational approaches to knowledge production through engaging relevant experts in open ‘talanoa’ conversations from an early stage [32]. This helped to mitigate the limitations of conventional global health evidence reviews, which privilege interventions documented in academic publications. Because much Pacific VAW/C prevention work is held in community and organisational knowledge rather than formal literature, these conversations helped us identify interventions that would otherwise be difficult to locate and contributed to a broader and more contextually grounded mapping of the evidence. This systematic scoping review was conducted in two complementary stages: 1) systematic literature searching and 2) expert consultation. We used the PRISMA-ScR extension to guide the process.

### Systematic literature searching

**Fig 1:**
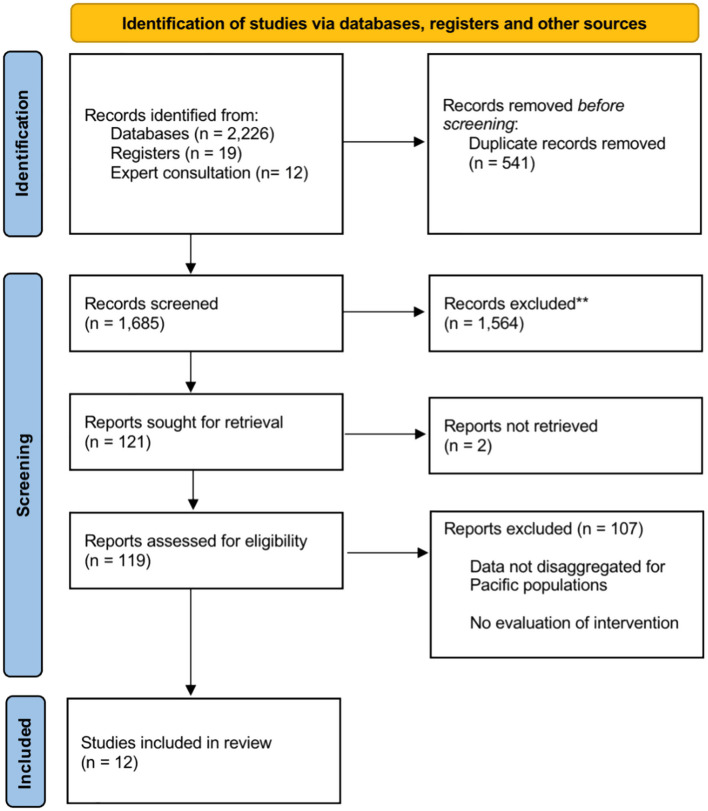
PRISMA flow diagram.

Firstly, we conducted academic database and grey literature searching to identify relevant studies. We ran searches in Embase, Medline, Global Health, PsycINFO and ProQuest in both English and French in June 2023 and then ran updated searches in February 2025. Search terms pertained to three concepts: VAW/C, the Pacific Islands region, and intervention studies (S1 Appendix). Studies were eligible for inclusion in the review if they were conducted in a Pacific Island Nation or Territory or with a Pacific Island population in Aotearoa New Zealand or Australia from the year 2000 onwards. We imposed no restriction on intervention type or study methods. Interventions had to be targeting either VAW, VAC or both VAW and VAC together. Intervention evaluations had to report on the impact of the intervention on either direct violence outcomes (e.g., prevalence of IPV, number of cases of child neglected reported to social services) or other proxy measures, such as those of gender norms and attitudes towards violence (see S2 Appendix for full inclusion criteria). Title and abstract screening was completed first by one reviewer, JU, who reviewed the titles and abstracts against the inclusion criteria to assess their conceptual relevance. A random selection of the included and excluded articles were then screened by a second reviewer, HL, who also completed full-text screening. During this second stage of screening, full texts were reviewed against the full inclusion and exclusion criteria. The most common reason for exclusion at this stage was that studies did not report an evaluation of a defined intervention, despite often being conceptually relevant. A third reviewer, JM, screened all articles retrieved in French, and a fourth reviewer, RM, conducted grey literature searching in Google, WHO Reproductive Health Library, UNICEF, UNDP and UNFPA regional webpages, UN Women Asia Pacific, Refworld, the Secretariat of the Pacific Community (SPC) and Toksave Pacific Gender resource. The same inclusion criteria was applied to the studies identified through the grey literature. After deduplication and two stages of screening, we included n = 12 VAW/C prevention intervention studies evaluating nine different interventions (Fig 1).

### Expert consultation

To enhance the systematic literature search and ensure comprehensive coverage of available studies, we conducted expert consultations to identify any relevant intervention studies that may have been overlooked. This approach was particularly valuable given that many interventions in the region are reported in the grey literature, and that substantial programming occurs outside academia, including within law and justice, advocacy, policy and faith-based sectors. Leveraging the authors’ existing networks, we invited stakeholders working on VAW and/or VAC in the Pacific region to participate in semi-structured interviews via Zoom. Three experts participated in interviews, while two additional stakeholders shared relevant resources via email. Experts included academics, staff working at multilateral agencies, and individuals working at multi-country Pacific organisations. Interviews were conducted between 1^st^ December 2024 and 31^st^ January 2025, audio-recorded, and transcribed. Ethical approval for this component of the study was obtained from University College London Research Ethics Committee. All interviewees gave written informed consent to participate. Three of the experts who contributed to study identification also joined the study team as co-authors on the manuscript, providing critical contextual understanding, framing and interpretation of the identified resources within the broader Pacific VAW/C prevention space. The expert consultations were instrumental in extending the scope of the review beyond the published literature, including identifying ongoing interventions and those with evaluations that are not publicly available. Through this process, two eligible interventions were identified that had not been captured through database or register searches and were subsequently included in the review.

### Data extraction and synthesis

Data on study characteristics were extracted into a pre-piloted form in Microsoft Excel by the first author, including on country of study, type of violence, study methods, sample size, analytical methods and findings. We also extracted data on the target population and intervention content, duration and outcomes. The extracted data for each intervention were then analysed thematically to explore common themes across the interventions, focusing primarily on the mechanism of intervention as a way to synthesise evidence across the diverse intervention types. We also explored pattens in the mode of intervention design and delivery, for example, whether interventions were co-designed with affected communities and to what extent Pacific worldviews were integrated into these processes. Results are presented thematically based on intervention type.

## Results

We identified eleven interventions that have been evaluated for their effectiveness in preventing VAW/C in the Pacific region, evaluated across five journal articles and nine grey literature reports. Nine interventions were identified through academic database and grey literature searching, and two interventions were identified through stakeholder consultation. We identified interventions in only five of the 22 Pacific nations included in this review, including the Solomon Islands, Samoa, Papua New Guinea, Fiji and Aotearoa New Zealand, (Table 1). Over half of the interventions targeted both VAW and VAC, three targeted only VAW, and only one intervention targeted solely VAC. We developed four overarching thematic categories to classify the main mechanism through which the interventions aimed to prevent VAW/C, including participatory learning and action (n = 2), community mobilisation (n = 3), community response (n = 2) and justice, policy and institutional response (n = 2). While some interventions operated across multiple levels, each was categorised based on its dominant methodology.

**Table 1:** Characteristics of studies evaluating interventions to prevent VAW/C in the Pacific region.

Characteristic	Interventions N (%)
**Total**	11 (100)
**Country**	
Solomon Islands	3 (27)
Samoa	2 (18)
Aotearoa New Zealand	2 (18)
Papua New Guinea	3 (27)
Fiji	1 (9)
**Type of violence targeted by intervention**	
Violence against children	1 (9)
Violence against women	3 (27)
Both	7 (64)
**Intervention type**	
Participatory learning and action	2 (18)
Community mobilisation	3 (27)
Community response	2 (18)
Policy and institutional response	4 (36)

Across the nine identified interventions (Table 2), the scope and detail of available evaluation data varied considerably. While six studies provided detailed insights into implementation processes and community engagement, five offered limited information about outcomes or mechanisms of change. This made it difficult to synthesise which approaches appear most effective in the Pacific context, likely reflecting broader challenges such as limited research capacity and evaluation funding in the region. Nevertheless, consolidating this dispersed evidence through a scoping review is valuable for highlighting emerging strengths, identifying key gaps, and informing future directions for VAW/C prevention research and practice in the Pacific.

**Table 2:** Details of evaluated interventions to prevent VAW/C in the Pacific region.

	Intervention	Country	Setting	Violence type	Intervention details	Intervention population	Evaluation methods	Impact
1	Kainga Tu’umalie Program	Aotearoa New Zealand (Tongan population)	Community (faith-based)	Family violence (VAW & VAC)	- Families from four church communities attended four weekend retreats - Formal activities included workshop sessions led by faith leaders on various topics related to family violence. Sessions used cultural practices *(Fofola e fala)* for collective Talanoa on causes and consequences of violence - Families also took part in informal activities including games, movies and sport	230 (49 families)	Qualitative (Talanoa with faith leaders and families)	- All families reported being positively impacted by the programme - Programme provided a space where children could openly share experiences, fathers could reflect on their use of violence and commit to a violence-free home, families could establish positive role models, parents could reflect on violent disciplinary practices and families could reflect on gender dynamics
2	Best Start, part of the Families Package	Aotearoa New Zealand (including Pacifica population)	Policy	Child abuse and maltreatment	- Unconditional cash transfers paid universally to mothers (or first caregiver) in the first year of child’s life	Approximately 13% of 173,169 infants	Quantitative (secondary analysis of administrative data)	- Families Package significantly reduced the odds of overall referrals to family services made by child protective services by 19% but the reduction observed in the Pacifica subgroup was not significant
3	Transforming communities to end sexual and gender-based violence	Solomon Islands	Community	Gender-based violence (VAW & VAC)	- Women volunteers from two provinces are trained to increase awareness of support services for survivors of violence and develop skills in basic counselling, paralegal and referral services. - Female volunteers form a committee and delegate responsibilities (providing paralegal support and referrals to other services and raising awareness of their services in the surrounding communities) - Committees work with service providers to establish referral protocols, raise awareness of GBV in schools and communities, and hold community awareness events	1,110 women and girls	Qualitative (interviews and FGDs)	- Increased awareness of GBV in the community and changes in attitudes of perpetrators - Some women reported reduced IPV - Reports of GBV to police and health workers increased - Greater involvement of village chiefs and church leaders in providing referrals and psychosocial support to survivors - Women felt empowered to advocate for the Family Safety Act
4	Oxfam Safe Families Programme	Solomon Islands	Community	Family and sexual violence (VAW & VAC)	- Community engagement facilitators (CEFs) trained to lead community engagement activities with support from local leaders - Family Violence Prevention and Response Action Committees (FVPRAC) of community members guided the implementation of activities - Activities for community members included: structured community conversations, workshops and edutainment on the topics of gender, power and violence - Provincial Alliance (PA) network of agencies established local referral system for survivors	6 communities in Temotu and Malaita provinces	Qualitative (in-depth interviews and FGDs)	- Increased awareness of VAW, some perpetrators reported using less violence, increased bystander intervention in communities - Positive changes amongst CEFs including increased confidence and greater understanding of harmful gender norms - Strengthened local response to family violence through the PA
5	Pilot Project to Increase Women’s Access to Justice in Guadalcanal and Malaita in Solomon Islands	Solomon Islands	Policy and legal	VAW	- Project worked with Authorised Justices (AJs) and Community Facilitators (CFs) - AJs established under the Family Protection Act (FPA) were granted powers to issue Interim Protection Orders (IPOs) for survivors of violence - CFs were trained and undertook awareness raising activities across their province on the role of AJs, GBV and their rights under the FPA	Communities across two provinces (Guadalcanal and Malaita)	Mixed methods (interviews and cross-sectional survey)	- Perception that there had been increased awareness of GBV and the FPA - Across the two provinces, 69% of women and 64% of men believed violence had stayed the same or increased - Increased number of IPOs issued by AJs during the project period
6	Advocacy for the Elimination of Violence Against Women	Papua New Guinea	Community, institutional/ government	VAW	- GBV training for local stakeholders, government officials, faith leaders and youth groups - Consultations with local leaders to understand priorities - Development of local bylaws and reporting and monitoring system to strengthen community level response to VAW	Jiwaka province	Mixed methods (semi- structured interviews, FGDs and cross-sectional survey)	- Voice for Change became a referral pathway between women and girls and the police and other services - Increased awareness of GBV - Resolution of ongoing tribal fighting - Reduction of VAW in public spaces (e.g., market)
7	Reporting, Investigating and Prosecuting Family and Sexual Violence Offences in Papua New Guinea	Papua New Guinea	Policy and legal	Family and sexual violence (VAW and VAC)	- System-level intervention focused on strengthening responses to family and sexual violence within policing and judicial processes - Examined implementation of laws and procedures related to reporting, investigation, and prosecution of offences - Included institutional reforms and practices such as Family and Sexual Violence Units (FSVUs), use of protection orders, and prosecutorial processes - Emphasised coordination between police, courts, and other justice actors	Not specified (system-level, including survivors engging with police and justice system actors)	Qualitative and mixed-methods (key informant interviews, case file reviews, policy and legal analysis)	- Increased institutional recognition of family and sexual violence and establishment of specialised units (FSVUs) - Ongoing constraints related to resources, capacity, and infrastructure - Attrition of cases across reporting, investigation, and prosecution stages - Barriers to accessing justice for survivors remain
8	Implementation of Family Protection Orders	Papua New Guinea	Policy and legal	Family and sexual violence (VAW and VAC)	- Civil protection order system introduced under the Family Protection Act (2013) - Includes interim protection orders (IPOs) and longer-term protection orders (POs) - Orders impose conditions on perpetrators to prevent further violence - Implemented through district and village courts as part of the formal justice system	118 protection order applicants across seven locations	Qualitative and mixed-methods (individual interviews with applicants, stakeholder interviews, analysis of administrative and justice system data)	- Increasing uptake of protection orders over time (prior to COVID-19 disruption) - Majority of applicants reported improved feelings of safety after obtaining orders - Protection orders linked to changes in living arrangements and separation from perpetrators - Ongoing challenges with enforcement, compliance, and access, particularly in rural areas - Implementation constrained by limited resources and broader justice system capacity
9	Preventing Violence against Women in Fiji’s Faith Settings	Fiji	Community (faith-based)	VAW	- Adaptation of the SASA! Faith intervention	3 Christian communities	Mixed methods (cross-sectional survey and interviews)	- Intervention led to positive changes in knowledge, attitudes and behaviours (reporting VAW to local leaders) from baseline to midline - Men reported positive changes in their lives and relationships
10	Village Family Safety Committee Pilot Project	Samoa	Community	Family violence (VAW & VAC)	- Establishment of Village Family Safety Committees (VFSC) - VFSC attend capacity building training to support them in raising awareness through local activities, developing local safety protocols for family violence, and making referrals to specialised services - Establishment and implementation of village bylaws	6 villages	Mixed methods (cross-sectional survey, interviews and focus group discussions)	- Communities involved reported reductions in family violence since the pilot began (e.g., 60% of VFSC members reported violence had decreased in their villages) - Qualitative data showed high support from communities for the project and examples of perpetrators of violence changing their behaviour
11	E le Sauā le Alofa	Samoa	Community	VAW	- Intervention co-design process with 30 community-based researchers (CBRs) - Piloting of six manualised co-designed activities across 10 communities with sessions on power and inequalities, healthy relationships, communicating effectively, positive parenting, livelihood generation and supporting survivors of violence.	10 villages (1 urban and 9 rural)	Mixed methods (cross-sectional survey, individual interviews and CBR workbooks	- Significant reduction in the prevalence of physical violence perpetration post intervention - Significant reductions in women’s agreement with statements around harmful gender norms - Community members reported taking part in actions to prevent family violence and local leaders took greater responsibility for ensuring the wellbeing of community members

### Participatory learning and action

Two of the interventions intended to achieve reductions in VAW/C through a participatory learning and action approach. These interventions used structured, participatory, group-based training to engage community members in a group setting in critical reflection, dialogue, and skills building activities. The **Kainga Tu’umalie Programme** was a group intervention with four Tongan church communities in Aotearoa New Zealand where 49 families attended four weekend retreats with formal and informal activities. Families took part in workshop sessions led by faith leaders that focused on reflecting on topics related to family violence, like the causes and consequences of family violence, through bringing together traditional cultural knowledge and Christian values in a supportive space [33,34]. Through qualitative evaluation, all families reported being positively impacted by the programme, with fathers having the opportunity to critically reflect on their behaviours that were driving violence in the family following open discussion with their Kainga (family) about the negative impact the violence was having. This emphasis on fostering critical reflection and dialogue around power and relationships aligns with global evidence on effective primary prevention of VAW, which identifies critical reflection as an important mechanism through which the attitudes and behaviours underpinning violence can begin to shift [12,35].

In Samoa, the **E le Sauā le Alofa** (Love Shouldn’t Hurt) programme also attempted to engage communities in critical reflection and action on VAW/C prevention through a community-led group-based intervention [36,37]. The pilot intervention was co-designed with 30 community-based researchers in a Participatory Community-led Intervention Development (PCID) cycle and then implemented in 10 villages across Samoa with adult men and women. Participants attended six participatory group-based sessions focussing on power and inequalities, healthy relationships, communication skills, positive parenting, livelihoods and supporting survivors in the community. Post intervention, a borderline significant reduction in men’s self-reported perpetration of physical (8.1 to 1.5%) IPV was observed, as was a significant reduction in women’s support for some statements around unequal gender roles in the family. In the qualitative evaluation, community members reported being positively impacted by the intervention, particularly with regard to the matai (local leaders) taking greater responsibility for community wellbeing and tackling the upstream drivers of violence like encouraging educational and evening prayer attendance. While both of these interventions provide promising positive results, they were both implemented on a relatively small scale (Kainga Tu’umalie included 49 families and E le Sauā le Alofa included 289 community members) and did not include comparison groups, limiting our ability to determine, according to global health evidence standards, whether the observed changes were attributable to the intervention, or whether the changes are likely to be sustained and generalisable to wider settings.

### Community mobilisation

Three of the interventions implemented at the community level attempted to prevent VAW/C through community mobilisation. This approach aims to shift harmful social norms and build collective action against violence at the community level, engaging a wide range of stakeholders and community members to build critical momentum and mass to foster awareness and shared responsibility for ending VAW/C across place-based communities. Community mobilisation has been widely adopted as a VAW prevention strategy in low- and middle-income countries worldwide, with well-documented examples including SASA! in Uganda and its adaptations to others contexts [12,38], and Stepping Stones in South Africa [39–41]. While community mobilisation interventions tend to be multi-component, their main focus is usually to build a critical mass of community members, leaders and stakeholders who are engaged, informed and empowered to actively challenge harmful norms, dismantle stigma and provide effective support for survivors, ultimately creating an environment in which violence is no longer tolerated.

**Transforming Communities to End Sexual and Gender-Based Violence** (SGBV) in the Solomon Islands used community mobilisation to build awareness, knowledge and skills among community members to respond to and prevent SGBV while also increasing access to essential services for survivors [42]. The 24-month project established a counselling, paralegal support and referral network of local community-based volunteers. These volunteers formed a committee and worked with service providers to establish referral protocols and hold events to raise awareness of SGBV in schools and communities. The intervention targeted four communities across two provinces, with 1,100 women and girl beneficiaries, and 156 service provider beneficiaries. The intervention was evaluated qualitatively through interviews and focus group discussions (FGDs) with primary and secondary beneficiaries and was believed to increase community awareness of SGBV, garner commitment from local leaders to support survivors, and empower and build capacity among the women volunteers. However, it also faced many challenges, including significant resource constraints and poor management.

The second community mobilisation intervention in the Solomon Islands, the Oxfam **Safe Families Programme**, took a similar multi-layered approach, training community engagement facilitators (CEFs) who led local awareness raising activities, developing community action plans for responding to violence locally, and developing provincial alliances (multistakeholder coalitions involving health services, police, women’s organisations, church groups, and daily support centres) to share information and plan collective actions to strengthen local services and responses to VAW/C [43]. Qualitative evaluation of the three-year intervention showed early signs of change in the intervention communities, with increased awareness and bystander intervention, strengthening of local services through the provincial alliances, and the generation of positive role models in the community with the CEFs who built long-term trusting relationships with community members.

The final community mobilisation intervention, an adaptation of **SASA! Faith**, was implemented in Fiji by the non-governmental organisation House of Sarah.[44] The intervention engaged religious leaders and community members throughout three Christian communities across four phases: Start, Awareness, Support and Action, delivering specific training and activities to foster non-violence and equality. The limited evaluation data available for SASA! Faith in Fiji (baseline survey n = 40 and midline survey n = 60) indicates early signs of positive change, including attitudes becoming less supportive of VAW/C and indications of behaviour change amongst men, for example *“If things were not followed, I’d beat my wife up. I was harsh on the kids. That’s how I disciplined my family. When the project came, I attended sessions given by community activists. I learned a lot there. I began to change the way I think. I needed to stop what I was doing. I became a member of the Men’s Group. Now, I share my story with other men in my community.”* However, further information is needed to understand the mechanisms through which this kind of change was brought about.

### Community response

Two of the included interventions attempted to prevent VAW/C through strengthening local capacity to respond to and prevent violence. Community response interventions involve establishing village- or community-level governance structures to implement local policies and bylaws, increasing local level ownership and accountability for the problem. The **Village Family Safety Committees Pilot Project** in Samoa aimed to use a culturally responsive approach to empower six Samoan villages to take a leading and proactive role in responding to family violence through the formation of Village Family Safety Committees (VFSC) [45]. The committees role was to raise awareness of family violence in the village, develop local action plans for responding to cases of violence, and refer survivors to specialised services. Differing from the community mobilisation interventions, the VFSC project focused solely on institutionalising a formal community-led governance structure, rather than building grassroots momentum and advocacy to oppose VAW/C. The qualitative evaluation of the VFSC project found that the two-year pilot project was successful in three of the six implementation villages, with nearly a quarter of village member participants saying they had reduced or stopped perpetrating violence since the programme, for example, *“I’m happy with the change now of being able to talk things out with my wife rather than resorting to violence and beating her up”*. The VFSC programme, supported by the UN Spotlight Initiative and the Samoa Office of the Ombudsman/ National Human Rights Institute was also a small-scale pilot project without a comparison group.

**Advocacy for the Elimination of VAW** in Papua New Guinea was a three-year, multi-component initiative combining local policy development with community education in Jiwaka, a newly established province (2012) [46]. The intervention aimed to increase accountability and action among provincial and local governments, community leaders, faith groups, and civil society to promote gender equality and protect women’s rights. It sought to strengthen local legal frameworks and improve responses to VAW by conducting community consultations, delivering gender and human rights training to diverse stakeholders (from local government officials to youth leaders), drafting and advocating for new local bylaws, developing a network of community advocates, launching media campaigns, and establishing local reporting and evaluation systems. This intervention was highly context-specific to this newly established province in Papua New Guinea where there were opportunities for communities to actively contribute to establishing new local governance mechanisms that were supportive of ending VAW and promoting women’s rights. They also included education campaigns and developed a network of community advocates, sharing some similar elements with community mobilisation interventions. Qualitative evidence suggests that the intervention led to perceived reductions in public daytime violence, including safer markets for women vendors and decreased street harassment. Some community members also reported reductions in IPV and domestic violence cases reaching village courts. These achievements were promising despite significant challenges, including weak provincial government capacity, logistical difficulties in rural areas, and resistance to shifting deeply engrained cultural and gender norms.

### Justice, policy and institutional response

The final four interventions included in this review focused on changing or implementing formal justice systems, laws or policies to address violence at the structural level, attempting to strengthen the broader institutional environment for preventing and responding to VAW/C. The first policy level intervention targeted VAC alone through an evaluation of **Best Start**, an unconditional cash transfer in Aotearoa New Zealand [47]. Implemented for babies born between April 1 and July 1, 2018, it provided NZ$60 per week to all parents in the first year and continued in years two and three for families below the 65th income percentile. Part of the *Families Package*, its goals were to reduce child poverty, improve child well-being, and give parents more flexibility in work and caregiving. A difference-in-difference analysis assessed its impact on referrals to family services, urgent child protection cases, and neglect/abuse. Overall, the analysis estimates that Best Start reduced child protective service referrals by 19%, driven primarily by decreases for Māori children, with no significant effect for Pacifica children.

The second intervention, **Increasing Women’s Access to Justice**, was a pilot project in the Solomon Islands aimed at advancing the *Family Protection Act 2014* [48]. It established *Authorised Justices* (AJs) – local court justices empowered to issue *Interim Protection Orders* (IPOs) for family violence – and introduced *Community Facilitators* (CFs) to raise awareness about AJs, legal rights, and VAW/C issues. Over three years, the project engaged 46 AJs in 37 communities and 40 CFs in two provinces. While IPO issuance increased, quantitative surveys in beneficiary communities found minimal changes in attitudes, understanding, or responses to VAW/C. By endline, 69% of women and 64% of men felt violence had worsened or remained unchanged in their community, and community engagement with AJs and CFs was low.

Two additional justice, policy and institutional interventions were identified in Papua New Guinea. The first, **Reporting, Investigating and Prosecuting Family and Sexual Violence Offences in Papua New Guinea**, examined reporting, investigation and prosecution of family and sexual violence offences within formal justice systems in Papua New Guinea [49]. Rather than evaluating a single programme, this study focused on system-level reforms to policing and judicial processes, including the establishment of Family and Sexual Violence Units (FSVUs), use of protection orders, and changes to investigative and prosecutorial practices. Using qualitative and mixed-methods approaches (including key informant interviews, case file reviews, and policy and legal analysis), the study found increased institutional recognition of family and sexual violence and the development of specialised response structures. However, implementation challenges remained, including limited resources, capacity and infrastructure, as well as substantial attrition of cases across reporting, investigation and prosecution stages. Barriers to accessing justice for survivors remained, alongside ongoing challenges in coordination between police, courts and other justice actors.

The second justice sector intervention in Papua New Guinea evaluated the **implementation of Family Protection Orders (FPOs)** under the Family Protection Act (2013) [50]. This legal mechanism provides interim protection orders (IPOs) and longer-term protection orders (POs) issued through district and village courts, aimed at preventing further violence. Based on interviews with applicants and stakeholders, as well as analysis of administrative and justice system data, the study found increasing uptake of protection orders over time (prior to COVID-19 disruptions). Many applicants reported improved feelings of safety following issuance of orders, with some describing associated changes in living arrangements and separation from perpetrators. However, the study also identified ongoing challenges in enforcement and compliance, particularly in rural areas, as well as limited access to services. Overall, implementation was constrained by broader justice system capacity and resource limitations.

### Promising intervention elements in the Pacific context

The Pacific cultural context was integrated into the design and delivery of the majority of the included interventions. While in-depth understanding of the unique mechanisms through which these approaches achieve impact is missing from the evidence base, a number of the interventions did reflect on their potential. Two of the interventions were faith-based, meaning that they leveraged religious institutions, leaders and beliefs to address VAW/C. A faith-based approach is particularly significant in the Pacific Islands context as religion plays a central role in governance, social structures and daily life, with most Pacific nations having a majority Christian population. SASA! Faith, implemented in Fiji, engages religious leaders to speak out against VAW, reinterpret religious texts to promote nonviolence and gender equality, and become community role models, engaging community members in dialogue and action through faith groups [44]. While extensive evaluation of the the mechanisms through which faith-based approaches can leverage success in VAWG interventions was lacking across the included studies, authors of the *Fofola e Fala* evaluation in Tonga argued that the church community provided a key mechanism through which positive messages and support for families can be spread and enhanced to increase intervention success [33]. Similar approaches have shown promise elsewhere, with faith-based organisations increasingly recognised as critical partners in responding to VAW/C. For example, in the Democratic Republic of Congo, the Transforming Masculinities intervention trained faith leaders and community groups to challenge gender inequality and promote nonviolence, resulting in reported reductions in IPV and improved gender attitudes [51].

Another promising element of a small number of interventions was their *culture as treatment* approach, which leverages local knowledge, cultural values and community practices to create more acceptable and sustainable change. The *Fofola e Fala* intervention with Tongan communities in Aotearoa New Zealand foregrounded the importance of Tongan family structures and worldviews in the design and delivery of their family violence intervention, avoiding repeating Eurocentric models which tend to be individualistic and focus on nuclear family structures [33]. One of the main intervention activities was the *Fofola e Fala* sessions (laying out the fine mat) in which family members were encouraged to discuss openly together different topics relating to family violence. This strengths-based approach to engagement, embedded in Tongan traditions and identity, supported families to “suspend social hierarchies”, create a safe space for sensitive discussions, and use existing cultural protocols in coming up with solutions to address family violence [34]. The authors conclude that it is the embedding within a Tongan worldview that led the intervention to resonate so strongly with participating families, contributing to its success [34]. The VFSC pilot project in Samoa also used existing community strengths to address VAW/C, drawing upon local governance structures to strengthen mechanisms for community response and prevention of VAW/C [45].

Finally, the E le Sauā le Alofa intervention in Samoa took a co-design approach to involving community members in intervention design and delivery, which is particularly salient in the Samoan context given its colonial history and negative legacies of extractive research [52]. Community-based researchers participated in iterative cycles of testing, modifying and implementing intervention activities that they felt were inherently valuable for their community [53]. The authors argue that co-design processes like this not only create spaces for communities to identify their own solutions to well established problems for greater relevance and sustainability of interventions, but also work towards greater epistemic justice. This aligns with growing momentum to decolonise VAWG research by centring local knowledges, redistributing power in evidence generation, and scrutinising the ways in which historical and contemporary colonial practices continue to shape the field of VAWG prevention [54].

### Barriers to intervention success

Despite these promising elements for intervention design in the Pacific, a number of cross-cutting barriers limited the extent to which interventions were able to achieve sustained impact. Many interventions were constrained by the resources and capacities required to implement them effectively. For example, community mobilisation and participatory learning interventions, including the Oxfam Safe Families programme in the Solomon Islands [55], relied heavily on facilitators and ongoing community engagement, yet reported challenges in maintaining participation and translating dialogue into sustained action. Short implementation timeframes and limited follow-up further constrained the depth of change achieved. Across multiple studies, geographic dispersion and the logistical challenges of working in rural and remote settings also limited reach and continuity, particularly where programmes depended on regular in-person engagement and took place across dispersed island or rural communities.

Similarly, while culturally grounded and community-responsive approaches were identified as a key strength, deeply embedded gender norms and social hierarchies continued to shape the extent to which change could occur. Evidence from faith-based and community interventions in Fiji and the Solomon Islands highlighted that increased awareness and dialogue did not consistently translate into behavioural change, particularly where women’s ability to act remained constrained by social and structural factors [46]. In parallel, justice, policy and institutional responses, including efforts to strengthen Family Protection Orders and policing and prosecution processes in Papua New Guinea, and the Increasing Women’s Access to Justice initiative in the Solomon Islands, were similarly shaped by broader system limitations [49,50,56]. While these interventions demonstrated progress in strengthening formal mechanisms (e.g., increased issuance of protection orders or establishment of specialised units), they were constrained by limited resources, weak enforcement and compliance, and ongoing barriers to access, particularly in rural areas.

Across the evidence base, there was also limited examination of mechanisms of change, making it difficult to assess how key intervention components, such as community participation or system strengthening, translated into outcomes.

## Discussion

This is the first review, to our knowledge, to synthesise evidence on prevention interventions for VAW/C in the Pacific region. We found a small but growing evidence base. The majority of the interventions targeted either VAW or family violence (VAW and VAC) – there is extremely limited evidence for interventions targeting VAC alone in the Pacific region. Similarly, the majority of interventions focused on preventing IPV, with no evidence on the broader types of VAW like non-partner sexual abuse and other gendered forms of violence.

The gaps in evidence for VAC in the Pacific region are stark – almost no interventions had a primary focus that included preventing VAC in any of its diverse forms. This likely reflects a combination of factors, but an important one to note is the lack of prevalence data on VAC in the Pacific region, which is essential for drawing attention to the magnitude of the issue and advocating for greater resources and prioritisation on the global health agenda. The global implementation of Violence Against Children and Youth Surveys (VACS), led by Together for Girls, have led to unprecedented levels of new information on the types, prevalence, settings and perpetrators of VAC in over 20 Global South countries [57]. They are a critical resource that has driven programme implementation and policy change across Africa, Asia, the Caribbean and Latin America [58], yet to this date, not a single VACS survey has been conducted in any Pacific country. Without vital prevalence data, it remains difficult to build the political momentum, allocate targeted funding, or design the context-specific interventions that are needed to address VAC as a public health and human rights priority in the region.

Evidence for the prevention of VAW in the Pacific region is growing, alongside significant political will and resource prioritisation, but more work is needed. There are promising results from participatory learning and action and community mobilisation interventions focused on intimate partner and family violence prevention, particularly those that were grounded in Pacific frameworks and values, employing strengths-based, culturally nuanced, faith-based and co-designed approaches [43,34,42,44,45]. These modes of intervention delivery have been shown to be effective in other contexts worldwide [12], but further research is needed to understand how they facilitate change in Pacific contexts. For example, interventions implemented by organisations that are known for their cultural strengths or religious frameworks have been critiqued by Pacific scholars as trying to prevent violence while at the same time reaffirming the unequal gender dynamics that drive it [59,60]. This raises the complexity and potential conflict between upholding Indigenous and religious rights, and the prevention of gendered forms of violence [52]. Further research is needed in the Pacific to fully understand how these discourses interact with violence prevention interventions across different contexts, and whether they serve to undermine or support current efforts.

Similarly, justice, policy and institutional level response interventions demonstrate early positive results, but there’s also a need to consider possible negative unintended consequences of policy change interventions when used alone and not alongside community-oriented social and behaviour change work at the individual and community levels [61,62]. The fact that the existing evidence on VAW prevention in the Pacific is centred around a very small handful of Pacific countries/populations poses a significant challenge. The scarcity of research not only hampers the identification of effective solutions, but also reflects the broader issue of the Pacific’s exclusion from global health initiatives. Given the diverse nature of the Pacific region, and the high and variable prevalence of VAW across individual Pacific countries, funding needs to be targeted towards scaling interventions to more Pacific populations. This necessitates a “deep adaptation” of interventions for each individual country (and often island) context, acknowledging local knowledge systems and epistemologies derived from three millennia of cultural history as part of intervention adaptation [63]. This would serve the dual purpose of building a global evidence base of how to adapt VAW prevention interventions that are relevant for Oceanic communities (supplementing current global efforts [64], while helping to improve the lives of women and children in the region.

Evidence that this is not happening arises from the consistent limitations across many of the interventions in both scale and duration. The dominant framework for global health research and practice provides strict and time bound funding, requiring research findings to be published within short funding periods. Many studies report on small-scale pilots with limited follow-up, leaving significant gaps in our understanding of sustainability and scalability. This points to an urgent need for greater investment in human capacity development in research and evaluation across the region. Moreover, building stronger, reciprocal partnerships between researchers, policymakers, and practitioners is essential. Research should not exist in a vacuum – it must be designed with clear pathways to influence programming and policy, just as programming should embed robust evaluation mechanisms from the outset.

A significant limitation of our review is the use of a global/public health framing of what constitutes ‘evidence’ in VAW/C prevention. Conceptualising evidence as programme evaluations that assess whether or not an intervention has reduced VAW/C outcomes might be contradictory to Pacific understandings, which may draw on other outcomes to assess the success or failure of interventions, such as community ownership, engagement and participation. In focusing on the evaluation of VAW/C outcomes, we may have missed critical information from VAW/C prevention in the region that use alternative methods for evaluating and sharing findings from programmes and interventions. This highlights a broader need for alternative approaches to evaluating interventions and to scoping evidence in global health. Further exploration into approaches to evaluation that draw on Pacific epistemologies and that can advance understanding of impact, scale and sustainability is warranted. Pacific-centred evaluation approaches, such as the Pacific Monitoring and Evaluation Framework, which emphasises Pacific values, relationships and priorities in measuring ‘success’ have the potential to go beyond traditional post-positivist and Global North interpretations of what successful interventions should look like [65].

While our approach to mapping the evidence has limitations, we attempted to overcome some of these challenges through our inclusion of stakeholder consultation in the evidence retrieval process. The opportunity to speak with scholars and practitioners working in the Pacific on VAW/C prevention was deliberately aligned with the Pacific value of *relationality* (through continuous conversations about interventions and their meaning; reciprocated and fostered through joint authorship) [32], and proved to be an invaluable exercise not only in identifying existing and ongoing work in the region, but in better understanding the breadth and depth of work taking place, the vast network of dedicated individuals and organisations involved, and the political, financial and human resource barriers that exist in the process of implementing, evaluating, and publishing evidence on interventions to instigate policy and social change. Their expertise strengthened the review by situating the synthesis within local prevention practice and ensuring cultural and regional relevance in the interpretation and dissemination of the evidence.

## Conclusion

This is a pivotal moment for the prevention of violence against women and children in the Pacific region, marked by growing political will, increased investment, and expanding programmatic efforts. Despite persistent evidence gaps, the breadth of existing initiatives offers substantial promise. With sustained resource allocation, strengthened local capacity, and coordinated implementation of evidence-informed programmes and research and evaluation, there is a clear opportunity to drive meaningful and lasting reductions in violence across Pacific communities.

## Supporting information

S1 Appendix Search terms and example search string. (DOCX)

S2 Appendix Review inclusion criteria. (DOCX)

S1 Checklist PRISMA checklist. PRISMA-ScR Checklist. Reproduced from the PRISMA Extension for Scoping Reviews (PRISMA-ScR), licensed under CC BY 4.0 (https://creativecommons.org/licenses/by/4.0/).
(DOCX)
