# Supplementary material for: Violence against women and children in the Pacific: A systematic scoping review and expert consultation of prevention interventions
Source: PLOS Glob Public Health. 2026 Jul 2;6(7):e0006587. doi: 10.1371/journal.pgph.0006587 (PMC13327266; doi:10.1371/journal.pgph.0006587)
Supplement: S1 Appendix — (DOCX) [file pgph.0006587.s001.docx]

**S1 Appendix**

**Search terms**

|  | **Search concept** | | |
| --- | --- | --- | --- |
|  | **VAW/C** | **Interventions** | **Pacific Island Countries** |
| **English terms** | Violence against women and girls OR VAWG OR VAW OR violence against women OR domestic violence OR domestic abuse OR spouse abuse OR intimate partner violence OR IPV OR family violence OR sexual violence OR economic abuse OR violence against children OR child* abuse OR VAC OR corporal punishment OR school-based violence OR school violence | Intervention* OR program* OR evaluat* OR quasi-experiment* or experiment* or RCT or controlled trial or effectiveness or effect* or decrease* or improve* or reduc* or comparative study or treatment* or service OR prevent* OR promot* OR campaign OR community OR school OR hospital | American Samoa OR Aotearoa OR New Zealand OR Cook Islands OR Federate States of Micronesia OR Fiji OR French Polynesia OR Guam OR Kiribati OR Marshall Islands OR Nauru OR New Caledonia OR Niue OR Northern Mariana Islands OR Palau OR Papua New Guinea OR Pitcairn Islands OR Samoa OR Solomon Islands OR Tokelau, Tonga OR Tuvalu OR Vanuatu OR (Wallis and Futuna) |
| **French terms** | La violence a l'egard des femmes et des filles or la violence contre les femmes OR violence familiale or violence domestique or la violence conjugale or violences sexualles or abus economique or violence contre les enfants or abus sur mineur or chatiment corporel or violence a l'ecole | Intervention OR programme OR evaluation OR quasi experimental OR experience OR essai or ECR OR essai controle randomise OR efficacite OR effet OR efficace OR diminuer OR ameliorer OR reduire OR reduction OR etude comparative OR traitement OR soins OR service OR prevenir OR la prevention OR promotion OR encourager OR campagne OR communaute OR ecole OR hopital | Samoa americaines OR Aotearoa OR Nouvelle Zelande OR les Iles Cook OR Etats Federes de Micronesie OR Fiji OR Polynesie francaise OR Guam OR Kiribati OR Iles Marshall OR Nauru OR Nouvelle Caledonie OR Niue OR Iles Mariannes du Nord OR Palaos OR Papouasie Nouvelle Guinee OR Iles Pitcairn OR Samoa OR Les iles Salomon OR Tokelaou OR Tonga OR Tuvalu OR Vanuatu OR Wallis et Futuna |

**Search sting in Ovid Medline**

1. (violence against women and girls OR VAWG OR VAW OR violence against women OR domestic violence OR domestic abuse OR spouse abuse OR intimate partner violence OR IPV OR family violence OR sexual violence OR economic abuse OR violence against children OR child* abuse OR VAC OR corporal punishment OR school-based violence OR school violence).ti,ab.
2. (intervention* OR program* OR evaluat* OR quasi-experiment* OR experiment* OR RCT OR controlled trial OR effectiveness OR effect* OR decrease* OR improve* OR reduc* OR comparative study OR treatment* OR service OR prevent* OR promot* OR campaign OR community OR school OR hospital).ti,ab.
3. (American Samoa OR Aotearoa OR New Zealand OR Cook Islands OR Federated States of Micronesia OR Fiji OR French Polynesia OR Guam OR Kiribati OR Marshall Islands OR Nauru OR New Caledonia OR Niue OR Northern Mariana Islands OR Palau OR Papua New Guinea OR Pitcairn Islands OR Samoa OR Solomon Islands OR Tokelau OR Tonga OR Tuvalu OR Vanuatu OR Wallis and Futuna).ti,ab.
4. 1 AND 2 AND 3
5. limit 4 to (humans and yr="2000 -Current")
