# Supplementary material for: Violence against women and children in the Pacific: A systematic scoping review and expert consultation of prevention interventions
Source: PLOS Glob Public Health. 2026 Jul 2;6(7):e0006587. doi: 10.1371/journal.pgph.0006587 (PMC13327266; doi:10.1371/journal.pgph.0006587)
Supplement: S2 Appendix — (DOCX) [file pgph.0006587.s002.docx]

**S2 Appendix**

**Inclusion criteria**

1. Intervention conducted in a Pacific Island Country or Territory as defined by the Secretariat of the Pacific community (SPC) or with a Pacific Island population in Aotearoa New Zealand or Australia
2. Conducted from the year 2000 onwards
3. Any intervention type (including primary, secondary and tertiary prevention)
4. Intervention must be targeting either VAW, VAC or VAW and VAC together
5. Any evaluation methods (qualitative, quantitative, mixed)
6. Evaluations must report on the impact of the intervention on either direct violence outcomes (e.g. prevalence of IPV, number of cases of child neglected reported to social services) or other proxy measures, such as those of gender norms and attitudes towards violence
7. Any publication type (journal article, grey literature report, thesis etc.)
